# Host-Directed Therapies for Cutaneous Leishmaniasis

**DOI:** 10.3389/fimmu.2021.660183

**Published:** 2021-03-26

**Authors:** Fernanda O. Novais, Camila Farias Amorim, Phillip Scott

**Affiliations:** ^1^ Department of Microbial Infection and Immunity, College of Medicine, The Ohio State University, Columbus, OH, United States; ^2^ Department of Pathobiology, School of Veterinary Medicine, University of Pennsylvania, Philadelphia, PA, United States

**Keywords:** cutaneous leishmaniasis, host-directed therapies, skin immunity, immunopathology, cytokines

## Abstract

Cutaneous leishmaniasis exhibits a wide spectrum of clinical presentations from self-resolving infections to severe chronic disease. Anti-parasitic drugs are often ineffective in the most severe forms of the disease, and in some cases the magnitude of the disease can result from an uncontrolled inflammatory response rather than unrestrained parasite replication. In these patients, host-directed therapies offer a novel approach to improve clinical outcome. Importantly, there are many anti-inflammatory drugs with known safety and efficacy profiles that are currently used for other inflammatory diseases and are readily available to be used for leishmaniasis. However, since leishmaniasis consists of a wide range of clinical entities, mediated by a diverse group of leishmanial species, host-directed therapies will need to be tailored for specific types of leishmaniasis. There is now substantial evidence that host-directed therapies are likely to be beneficial beyond autoimmune diseases and cancer and thus should be an important component in the armamentarium to modulate the severity of cutaneous leishmaniasis.

## Introduction

Cutaneous leishmaniasis is caused by several different species of protozoa transmitted by sand flies, and has a variety of clinical forms, ranging from self-healing lesions to chronic disfiguring mucosal disease (1, 2). There is no vaccine for the disease, and drug treatment is not always effective (3, 4). Moreover, in some forms of leishmaniasis the magnitude of the disease appears to be due to the uncontrolled inflammatory response at the cutaneous site of infection. It is clear that new therapeutic approaches are needed, and host-directed therapies to either enhance protective immune responses or to ameliorate excessive cutaneous inflammation represent novel therapeutic strategies worthy of pursuit.

Host-directed therapies for infectious diseases are designed to either amplify protective immune responses, divert non-protective immune responses towards protective responses, or block pathologic immune responses (5). Fortunately, our in-depth understanding of both protective and pathologic immune responses and identification of agents that can be used clinically to influence immune responses has revolutionized treatment of a wide range of diseases. While many of these new treatments are for non-communicable diseases, repurposing such treatments for infectious diseases, such as cutaneous leishmaniasis is advantageous, as their safety and efficacy profiles have often already been established.

In order to be successful, host-directed therapies must not overstimulate the immune response, or block protective immune responses necessary to control the pathogen. These are not theoretical possibilities. For example, checkpoint blockade has revolutionized cancer treatment, but some patients develop adverse events associated with these treatments, including cytokine storms that can be lethal (6, 7). Similarly, anti-inflammatory treatments run the risk of increased susceptibility to infections. Thus, the key to using host-directed therapy with infectious diseases is to lessen the chances of adverse events by defining the mechanisms mediating protection as well as those promoting immunopathologic responses associated with the disease. In cutaneous leishmaniasis there is a good understanding of the protective mechanisms, and thus one strategy is to promote those responses. Here we will review the host-directed therapies that could be used to enhance protection in patients. Many of the studies discussed focus on murine models where potential host-directed therapies can be assessed prior to initiation of clinical trials with patients.

We will also discuss what we know about destructive inflammation seen in patients with chronic cutaneous leishmaniasis and identify potential targets for therapies to promote disease resolution.

## Spectrum of Clinical Presentations in Cutaneous Leishmaniasis

A challenging aspect in lessening disease in cutaneous leishmaniasis is the variety of clinical presentations associated with the infection. The type of clinical presentation is driven by the nature of the immune response invoked, which is influenced by both host genetics and the specific species or strain of the parasite causing the infection (1, 2). Following infection by a sand fly, patients develop a small nodule which progresses to an ulcerated lesion that will eventually heal in several months. However, in some cases, the lesions fail to resolve, or the parasites spread to many cutaneous sites without any evidence of control, a form of leishmaniasis known as diffuse cutaneous leishmaniasis (DCL) (8–10). These patients fail to develop a delayed-type hypersensitivity response or a strong IFN-γ response, and thus parasite burdens in the lesions are extremely high (9, 10). Histologically, these lesions appear as masses of macrophages with large numbers of intracellular parasites, and few infiltrating lymphocytes (10). It is clear that enhancing a protective immune response would be important for this disease.

At the other end of the spectrum, parasites can spread to the naso-oropharyngeal mucosa and cause extensive damage mediated by an uncontrolled immune response. This disease, termed mucosal leishmaniasis, is most often caused by *L. braziliensis* parasites and is refractory to anti-parasitic treatment. While the parasites are largely controlled by the immune response, there is a large infiltration of inflammatory cells into the lesions, suggesting that the damage is due to an overexuberant inflammatory response rather than uncontrolled parasite growth (11). While mucosal leishmaniasis is the most severe form of the disease at the inflammatory end of the spectrum, single lesions in patients infected by *L. braziliensis* can also be chronic, resistant to drug treatment, and associated with a severe inflammatory response with a low parasite burden in the lesions.

Patients who fail to develop a protective Th1 cell response develop disease, often in spite of a strong antibody response. This is most clearly observed in DCL patients (9). In contrast, patients with a strong Th1 cell response also develop severe disease, but in this case due to inflammation rather than massive parasite numbers (11). This spectrum is not unique to cutaneous leishmaniasis. For example, in another cutaneous disease, leprosy, the disease ranges from lepromatous leprosy in which there is an absence of a strong T cell response and no control of the bacteria to tuberculoid leprosy in which bacteria are scarce, and the immune response causes disease (12, 13). Unfortunately, drug treatment for cutaneous leishmaniasis patients with severe disease at either end of the spectrum can be ineffective, which provides support for considering alternative treatment strategies (8, 10, 14). However, what is clearly evident is that any host-directed therapy will need to take into consideration where a patient is on this immunologic spectrum.

Experimental models of cutaneous leishmaniasis have been critical for understanding the disease, and important in defining the mechanisms associated with T cell subset development. For example, infection of mice with *Leishmania major* helped define the factors driving CD4 Th1 and CD4 Th2 cell development and maintenance (15–17). These studies established the critical role of IFN-γ produced by CD4 T cells in protection, and the lack of a protective role for antibodies. In contrast, infection of BALB/c mice with *L. major* results in an uncontrolled infection, which is in part due to the development of a Th2 response. While these uncontrolled infections mimic some aspects of DCL (or visceral leishmaniasis), the role of IL-4 in promoting increased disease in patients is less clear than in murine models (18). Many studies have been done with *L. major*, but these do not represent the whole breadth of disease patterns that can be seen with other species of *Leishmania*. For example, while C57BL/6 mice resolve disease following infection with *L. major*, lesions induced by either *L. amazonensis* or *L. mexicana* infections do not resolve (19, 20). In these cases, susceptibility is linked with the failure to develop a strong Th1 response, rather than a Th2 response. *Leishmania* strain differences can also influence disease outcome. For example, the *L. major* Seidman strain causes a non-healing infection in C57BL/6 mice in spite of the development of a Th1 response (21, 22). Although all murine models have their limitations, many of these different host-parasite models are useful to assess host-directed therapies that can enhance immune responses. In contrast, fewer models have been available that mimic the excessive inflammatory responses associated with patients infected with *L. braziliensis* parasites (see below).

## Enhancing Protection in Cutaneous Leishmaniasis by Host-Directed Therapies

*Leishmania* parasites replicate in myeloid cells, including macrophages, monocytes and dendritic cells. Control of the parasites is dependent upon activation of these cells by IFN-γ, leading to increased production of nitric oxide and/or reactive oxygen species, although the role of these molecules may vary depending upon the host and the parasite species (23–26). The primary source of IFN-γ that leads to protection in cutaneous leishmaniasis is the CD4 T cell, although CD8 T cells and NK cells can also contribute to protection (27–29). Once an infection has resolved, resident memory CD4 Th1 cells in the skin, central memory CD4 T cells and circulating effector CD4 Th1 cells maintained by persistent parasites provide protection against a secondary challenge (30, 31). Since resident memory Th1 cells can be maintained in the absence of persistent parasites, they are a good target for vaccine development. While we understand how the immune response can control these parasites, there are multiple mechanisms that can block or lessen the development of protective responses, which is why lesions often take so long to resolve. Defining these barriers to protection can provide targets for host-directed therapies in patients in whom limited Th1 responses develop.

**Figure 1:**
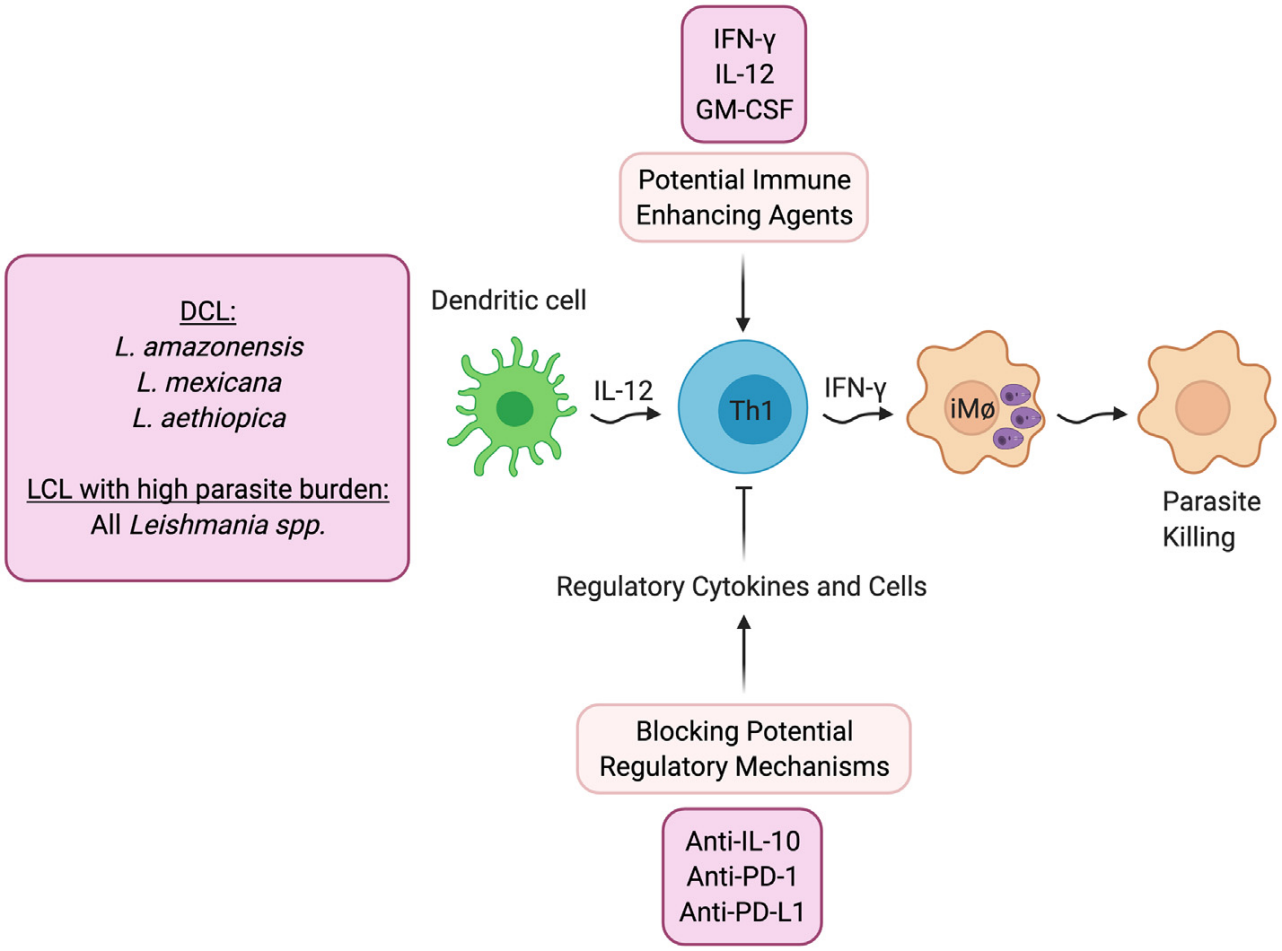
Host directed therapies that promote better parasite control. DCL- Diffuse cutaneous leishmaniasis; LCL- Localized cutaneous leishmaniasis.

A reasonable first line approach to promote healing is treatment with agents that directly increase protective immunity (**Figure 1**). One can define protective immunity in both experimental models and humans as the ability to protect against the development of disease, which may not lead to complete elimination of the parasites. While this protection may require IFN-γ, as discussed above it is also clear that IFN-γ by itself does not always lead to lack of disease.

As would be expected, treatment with IFN-γ has shown increased control in patients who are refractory to standard treatment (32, 33), and experimentally, IL-12 can promote healing even after lesions have developed when given in conjunction with an anti-parasitic drug (34, 35). In addition, clinical trials have been done with GM-CSF, in which topical treatment was found to promote increased healing (36, 37). Similarly, topical treatment with the TLR7 agonist imiquimod has shown increased healing rates (38), although there have been mixed results in clinical trials (39).

Alternatively, another potential therapeutic approach would be to block pathways that downregulate protective immunity (**Figure 1**). DCL patients fail to generate a protective IFN-γ response, and the pathology seen in these individuals is due to uncontrolled parasite growth in macrophages in the skin. While IL-4 blockade of protective responses can contribute to the uncontrolled *Leishmania* replication in experimental models, IL-4 appears to be less important in DCL patients (9) or indeed in any form of human leishmaniasis. Instead, a recent study suggests that DCL patients exhibit an overwhelming B cell response, and little evidence of either a Th1 or Th2 response (9). In contrast, IL-10 plays a critical role in promoting susceptibility to *L. major* in BALB/c mice, suggesting that blocking IL-10 might increase protective responses. Consistent with this possibility are studies in visceral leishmaniasis patients who can also develop uncontrolled infections. In these patients IL-10, rather than IL-4, has been linked with susceptibility. Importantly, a study with splenic aspirates from visceral leishmaniasis (VL) patients demonstrated that blockade of IL-10 enhanced control of the parasites (40), which provides the experimental foundation for a host-directed therapy where IL-10 would be blocked in VL patients (41). Experimentally, other regulatory cytokines have been shown to block protective Th1 responses in cutaneous leishmaniasis. For example, TGF-β inhibits protection in *L. amazonensis* infected mice (42), and IL-27 promotes IL-10 responses and increased susceptibility (43). Thus, blocking these regulatory pathways might promote better protective responses.

The role of inhibitory receptors in modulating the outcome of infectious diseases is an area of active investigation, since checkpoint blockade is effective in promoting control of cancer (44). One might predict, therefore, that blocking this regulatory pathway might be protective in cutaneous leishmaniasis as well. However, to date the experimental results in leishmaniasis are unclear. A study with arginase-deficient *L. major* in mice unable to resolve their infections found that anti-PD-1 monoclonal antibody promoted healing. However, blockade of PD-1 or PD-L1 in *L. amazonensis* infected mice (45) or infection of PD-L1 knockout mice with *L. mexicana* (46), had minimal effects on parasite control. A recent study found that T cells with an exhausted phenotype were present in the blood and lesions of *L. braziliensis* patients, and blocking PD-1 signaling in circulating T cells from patients enhanced their proliferation and production of IFN-γ (47). Clearly, more studies need to be done to understand the role of PD-1/PD-L1, as well as other checkpoint molecules, in cutaneous leishmaniasis.

## Controlling Immunopathology in Cutaneous Leishmaniasis by Host-Directed Therapies

Enhancing Th1 responses directly or blocking pathways that lessen Th1 responses will not be effective for every type of cutaneous leishmaniasis. This is particularly true for patients at the immunopathologic end of the spectrum who develop chronic lesions in spite of their ability to generate a strong Th1 response. This clinical presentation is best exemplified by *L. braziliensis* infections, where chronic lesions are associated with a strong Th1 response and few parasites. While IFN-γ and TNF are important for macrophage activation and parasite control, in excess both cytokines can be associated with pathologic immune responses and it is possible that a poorly regulated Th1 response leading to high levels of IFN-γ and TNF contributes to this chronic inflammation. Moreover, since blocking TNF is a successful host-directed therapy for patients with rheumatoid arthritis, it is reasonable to consider its role in blocking pathology in cutaneous leishmaniasis. In support, a recent study suggests that TNF in *L. mexicana* infections promotes T cell exhaustion (48). While clinical trials have not yet been done with humanized monoclonal antibodies against TNF, the drug pentoxifylin, which blocks TNF, has been used in *L. braziliensis*, but with mixed results (49–51).

The optimal pathway to target in patients at the inflammatory end of the spectrum would be one that is not associated with protection. Notably, studies in *L. braziliensis* patients uncovered a major pathway leading to disease that was independent of protective immune responses. These studies found that cytolysis by CD8 T cells correlated with increased pathology in cutaneous leishmaniasis patients (23, 52–61). Importantly, these studies were followed up with the demonstration that patients who eventually fail drug therapy can be identified prior to treatment based upon expression level of genes associated with cytotoxicity (59).

The identification of CD8 T cells as drivers of disease was initially confusing, since CD8 T cells were protective in models of cutaneous leishmaniasis. For example, infection of CD8 deficient mice with low doses of *L. major* leads to susceptibility (28). The protective role of CD8 T cells appears to be mediated primarily by promoting Th1 responses in the draining lymph nodes (27, 28). This paradox was resolved by the finding that CD8 T cells in the lesions made little IFN-γ, but were instead cytolytic (53, 54, 56). The mechanisms involved in the differential function of CD8 T cells in the draining lymph nodes and cutaneous lesions has yet to be understood, although one factor may involve the lack of local signals in the lesions that would promote IFN-γ production by CD8 T cells (62). These results raised the question of how cytolytic CD8 T cells promote disease in cutaneous leishmaniasis. Based upon other infections, one might predict that killing of *Leishmania*-infected cells would lead to better parasite control. However, the evidence suggests that instead of killing the parasites, lysing the infected cell results in parasite dissemination, which then go on to infect other cells (54). Thus, cytolysis may be one mechanism that promotes metastasis in patients.

As the pathologic role for CD8 T cells is difficult to ascertain in standard experimental models of cutaneous leishmaniasis new models to define the mechanisms leading to CD8 T cell mediated pathology needed to be created. The most straightforward model was the adoptive transfer of CD8 T cells into RAG mice followed by infection with *Leishmania* (28, 54). Importantly, RAG mice infected with *L. braziliensis* do not develop substantial lesions over many weeks of infection, in spite of a large number of parasites present at the site of infection (54). These results, and previous studies in RAG mice (63), demonstrate the critical role T cells play in developing ulcerated lesions. RAG mice receiving CD4 and CD8 T cells developed small lesions and controlled parasite replication. In contrast, RAG mice that received CD8 T cells alone and were infected with *L. braziliensis* developed severe uncontrolled lesions (54). Surprisingly, the number of parasites in infected RAG mice and RAG mice that received CD8 T cells was the same, highlighting the critical role for CD8 T cells in immunopathology. This CD8 T cell dependent pathology required the cytotoxic molecule perforin, but not IFN-γ, since transfer of perforin deficient T cells to RAG mice failed to induce pathology, while IFN-γ -/- CD8 T cells did (54). In a complementary model, bystander cytolytic CD8 T cells were also found to promote increased disease, as mice that had resolved an infection with lymphocytic choriomeningitis virus (LCMV) developed more severe disease than mice that had not previously been infected with LCMV in response to *Leishmania* challenge weeks after viral clearance (64, 65). In this model, LCMV specific NKG2D expressing CD8 T cells were recruited to the cutaneous lesions non-specifically and mediated killing of targets expressing NKG2D ligands that were upregulated on cells in the lesions due to inflammation. The relevance of bystander CD8 T cells to human leishmaniasis is suggested by the finding that lesions from patients who have been infected with *Toxoplasma* contained *Toxoplasma* specific CD8 T cells (66). Thus, studies in both experimental models, as well as gene transcriptional analysis of lesions from patients, identified an immunopathologic pathway dependent upon cytolysis in cutaneous leishmaniasis.

**Figure 2:**
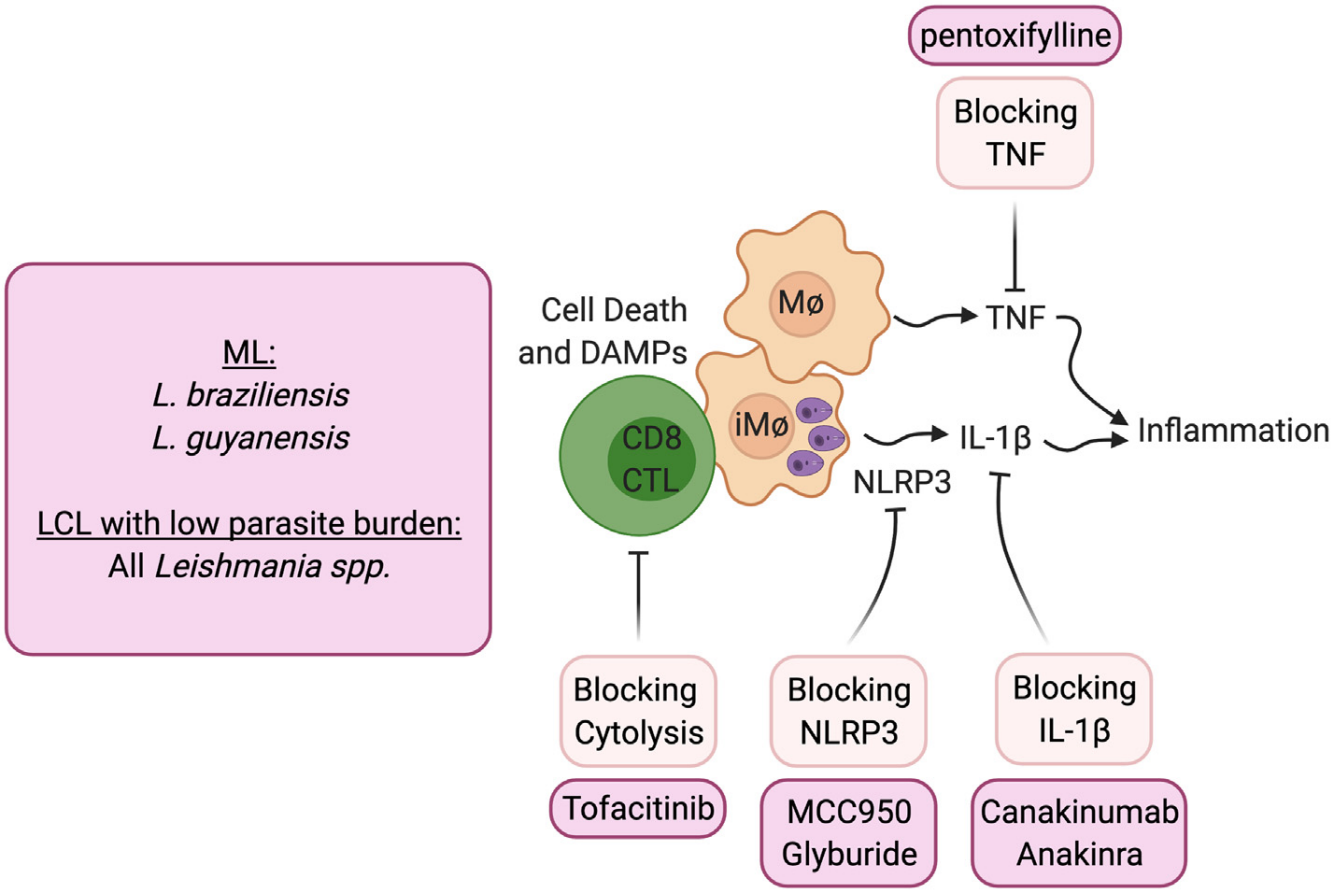
Host directed therapies that block immunopathologic mediated cytolysis. ML, mucosal leishmaniasis; LCL, Localized cutaneous leishmaniasis.

The transcriptional analysis of lesions from patients provided clues as to how cytolysis might promote increased disease (23, 67). Not only were genes associated with cytolysis upregulated in lesions, but those associated with inflammasome activation, including NLRP3, Caspase 1 and IL-1β, were similarly upregulated. The immunopathologic pathway hypothesized from gene transcriptional analysis of lesions was confirmed using the experimental models of CD8 T cell mediated disease described above. Thus, CD8 T cell mediated disease could be blocked by inhibitors of NLRP3, such as MCC950 and glyburide, or blockade of IL-1β with the IL-1 receptor antagonist anakinra or with anti- IL-1β antibody treatment (56) (**Figure 2**). The pathologic role for IL-1β is not limited to situations where there is uncontrolled CD8 T cell mediated cytolysis. Others have shown that inflammasome-dependent IL-1β mediates the severe disease seen with a virulent *L. major* strain, and IL-1β administration can exacerbate disease following *L. major* and *L. amazonensis* infection (22, 68, 69). In addition, IL-1 serum levels correlate with increased disease severity in *L. mexicana* patients (70), and more serious disease was reported in mice lacking the natural inhibitor of IL-1β signaling (IL-1RA) (71). In the *L. amazonensis* model IL-1β was found to promote resistance, although these mice fail to resolve with or without IL-1β (72). IL-1β has many roles in the immune response, but the pathologic role of IL-1β in cutaneous leishmaniasis appears to be when the cytokine is in excess. Notably, because both the inflammasome and IL-1β are associated with many chronic diseases, including autoimmune diseases, cancer and cardiovascular diseases, a number of inhibitors designed to block this pathway are in clinical use or are in clinical trials that can be tested in cutaneous leishmaniasis.

Blocking CD8 T cell cytotoxicity, an initiator of this pathway, could be another important target in lessening pathology. IL-15 is a potential target for such treatment, as it is highly expressed in lesions of human cutaneous leishmaniasis patients and promotes the expression of granzyme B dependent CD8 T cell cytotoxicity. Tofacitinib is a small molecule inhibitor of janus kinase (Jak)3 which is required for IL-15 signaling (73). It is currently being used clinically to treat certain types of arthritis under the trade name Xeljanz, and experimentally treats alopecia areata by blocking NKG2D dependent cytolysis (74). In experimental *Leishmania* models of CD8 T cell mediated pathology, systemic and topical treatment with tofacitinib blocked pathology (75). Notably, tofacitinib did not alter protective Th1 responses or parasite control. Thus, local targeting of CD8 T cell-mediated cytotoxicity can be a safe strategy to block immunopathologic responses locally while sparing protective responses.

## Conclusions

Host-directed therapies hold great promise for lessening the more severe forms of cutaneous leishmaniasis. The ease of monitoring the efficacy of host-directed therapies in cutaneous diseases is a significant advantage to such treatments, and particularly important is the potential to develop topical treatments that may reduce untoward systemic responses. While in many diseases host-directed therapies are administered systemically, for those that might be used in cutaneous leishmaniasis it will be important to test whether topical application might be effective. One successful experimental example is the treatment with tofacitinib, which we found was as effective at controlling disease given topically as given systemically (75).

It is evident that care must be taken in the development of such therapies, as there remains the potential for blocking a pathway critical for control of *Leishmania*. Importantly, all of these therapies should be used in conjunction with standard anti-parasite drug treatment which lessens the risk of unchecked *Leishmania* multiplication. While increased susceptibility to other pathogens might remain, the short treatment period required would also lessen this risk. Finally, a practical consideration for developing therapies for neglected tropical diseases, such as cutaneous leishmaniasis, is the cost of treatment. Clearly the utility of any new host-directed therapy will depend on cost. However, identification of the targets for a successful host-directed therapy is the first step and can provide the rationale for a search for cheaper alternative treatments targeting the same immunologic pathways.

With the seeming endless development of new approaches to modulate the immune response with cytokines, small molecule inhibitors, humanized monoclonal antibodies, and drugs directed against immune targets, there is a growing interest in applying host-directed therapies to infectious diseases. Cutaneous diseases, such as leishmaniasis, can clearly benefit from such treatments. However, the key to success will be a continued focus on understanding the mechanisms leading to protective and pathologic responses in the skin, where many unanswered questions remain to be addressed. Most studies of cutaneous leishmaniasis have focused on systemic responses, or those occurring in local lymph nodes, and have ignored the unique aspects of the skin. Differences in cell types, metabolism, oxygen levels, and temperature can influence the outcome of cutaneous leishmaniasis, but have been little studied in this disease. Further, the skin directly interacts with the external environment and the skin microbiome can have significant effects on the outcome of infection (76, 77). It is fair to say that the success of host-directed therapies for cutaneous leishmaniasis will depend upon a better understanding of the skin, and for leishmaniasis we have just “scratched the surface” in that arena.

## Author Contributions

FN and PS contributed to the conception and design of the review. FN, PS, and CA contributed to aspects of the review and writing. All authors contributed to the article and approved the submitted version.

## Funding

The authors were supported funding from NIH grants R01-AI-150606 and R01-AI-149456.

## Conflict of Interest

The authors declare that the research was conducted in the absence of any commercial or financial relationships that could be construed as a potential conflict of interest.
